# Antibiotic effects against periodontal bacteria in organ cultured tissue

**DOI:** 10.1002/cre2.48

**Published:** 2016-11-24

**Authors:** Masaaki Takeshita, Akira Haraguchi, Mayumi Miura, Takafumi Hamachi, Takao Fukuda, Terukazu Sanui, Aiko Takano, Fusanori Nishimura

**Affiliations:** ^1^ Section of Periodontology, Division of Oral Rehabilitation, Faculty of Dental Science Kyushu University Fukuoka Japan; ^2^ Division of General Dentistry, Kyushu University Hospital Kyushu University Fukuoka Japan

**Keywords:** antibiotic prophylaxis, intraepithelial infection, organ culture model, periodontal disease

## Abstract

Mechanical reduction of infectious bacteria by using physical instruments is considered the principal therapeutic strategy for periodontal disease; addition of antibiotics is adjunctive. However, local antibiotic treatment, combined with conventional mechanical debridement, has recently been shown to be more effective in periodontitis subjects with type 2 diabetes. This suggests that some bacteria may invade the inflamed inner gingival epithelium, and mechanical debridement alone will be unable to reduce these bacteria completely. Therefore, we tried to establish infected organ culture models that mimic the inner gingival epithelium and aimed to see the effects of antibiotics in these established models. Mouse dorsal skin epithelia were isolated, and periodontal bacteria were injected into the epithelia. Infected epithelia were incubated with test antibiotics, and colony‐forming ability was evaluated. Results indicated that effective antibiotics differed according to injected bacteria and the bacterial combinations tested. Overall, in organ culture model, the combination of amoxicillin or cefdinir and metronidazole compensate for the effects of less effective bacterial combinations on each other. This in vitro study would suggest effective periodontal treatment regimens, especially for severe periodontitis.

## 1 INTRODUCTION

Periodontal disease is a chronic inflammatory disease initiated by infection of several Gram‐negative anaerobic bacteria, and it leads to loss of attachment apparatuses to the root surface and surrounding tooth bone. Therefore, it is the main cause of tooth loss in the adults (Jeffcoat & Reddy, 1991). Additionally, it has been suggested that this disease induces systemic, low‐grade inflammatory responses in the host, which, in turn, induces undesirable influences on the systemic disease, such as type 2 diabetes (Madianos & Moutsopoulos, 2006).

*Porphyromonas gingivalis* (*Pg*) is the major periodontitis pathogens (Holt, Kesavalu, Walker, & Genco, 1999; Page, Offenbacher, Schroeder, Seymour, & Kornman, 1997). In addition, *Prevotella intermedia* (*Pi*) is reported to be resistant against penicillin and/or tetracycline, and it augments antibiotic resistance of other oral bacteria when co‐cultured with this microorganism (Andres, Chung, Roberts, & Fierro, 1998, Fosse et al., 2002, Takahashi, Ishihara, Kimizuka, Okuda, & Kato, 2006). Furthermore, *Fusobacterium nucleatum* (*Fn*) is one of the key microorganisms in forming bacterial biofilm, and it can aggregate with many other oral bacteria (Bolstad, Jensen, & Bakken, 1996).

Reduction of infectious bacteria via mechanical debridement, which is designated “scaling and root planing,” has been the principal therapeutic strategy for generic periodontitis subjects; addition of antibiotic therapy to mechanical debridement is considered adjunctive (Chei & Lu, 2005; Rabbani, Ash, & Caffesse, 1981). However, a recent report indicated that the combination of local antibiotic therapy with mechanical debridement resulted in greater reduction of systemic inflammatory responses, as assessed by high‐sensitivity C‐reactive protein and subsequent metabolic control, than conventional mechanical debridement alone in non‐obese subjects with type 2 diabetes complicated by severe periodontitis (Munenaga, Hiroshima Study, Yamashina, Tanaka, & Nishimura, 2013). Thus, it is quite possible that the subjects with diabetes are more susceptible to infectious bacteria although precise mechanism is still under investigation. Nevertheless, because it appears that local periodontal inflammation would be more clearly reflected in systemic inflammatory marker in non‐obese subjects than obese subjects (Borgnakke et al., 2014), the result suggests that mechanical debridement alone cannot effectively reduce infectious bacteria from periodontal lesions and bacterial infections extend not only to the gingival sulcus but also to the inflamed inner epithelium. In fact, it has been suggested that several periodontal bacteria, such as *Pg*, not only invade host tissue but also invade host cells (Lamont & Yilmaz, 2002; Takeuchi, Furuta, & Amano, 2011).

The effectiveness of antibiotic therapy in periodontal treatment has been reported by meta‐analysis (Sgolastra,Petrucci, Gatto, & Monaco, 2012; Zandbergen, Slot, Cobb, & Van der Weijden, 2013). Especially, the combination of amoxicillin trihydrate (AMX) and metronidazole (MTZ) appears to be highly effective by in vitro studies (Cionca, Giannopoulou, Ugolotti, & Mombelli, 2009, Ehmke, Moter, Beikler, Milian, & Flemmig, 2005, 2010; Feres et al., 2012, Goodson et al., 2012). Most of the previous studies evaluated the effects of antibiotics from clinical standpoint, and very few reports actually assessed antibiotic effects from systemic inflammatory responses. Few studies reported the effects of AMX and MTZ on response of inflammatory biomarker (Almaghlouth, Cionca, Cancela, Decaillet, Courvoisier, Giannopoulou, & Mombelli, 2014, Giannopoulou et al., 2016). Furthermore, the effect of the combination of AMX and MTZ on diabetes has also been reported (Miranda et al., 2014). However, most of these studies focused on the effects of one or two antibiotics, and these have not compared the effectiveness with other antibiotics. If combination of antibiotics with conventional therapy would result in greater reduction of systemic inflammatory response, it is essential to establish effective antibiotic strategies, especially for severe periodontitis subjects with impaired systemic conditions such as those with diabetes. To achieve this, we aimed to establish organ culture models that mimic the infected inner epithelium and then evaluate the effects of antibiotics against established infection models.

## 2 MATERIALS AND METHODS

### 2.1 Bacterial strains and antibiotics

The bacterial strains used in this study were *Pg* (W50), *Pi* (ATCC 25611), and *Fn* (ATCC 10953). Each strain was grown to the late log phase in α‐MEM (NACALAI TESQUE, Japan) containing 10% fetal bovine serum (FBS; Biowest, France) at 37 °C under anaerobic conditions (10% CO_2_, 10% H_2_, and 80% N_2_). Prior to the experiments, growth rate of tested bacteria in α‐MEM supplemented with FBS has been compared with the one in conventional brain–heart infusion medium, and no significant difference was observed in terms of growth rate (data not shown). Culture media were adjusted to optical density at 590 nm (OD_590_) of 1.0 for *Pg* and *Pi* (OD_590_ 1.0: 100 μl of log_10_ colony forming unit [CFU] = 9.0) and of 1.0 for *Fn* (OD_590_ 1.0: 100 μl log_10_ CFU = 7.0). OD_590_ was measured using a spectrometer.

The antibiotics used in this study were as follows: penicillin/streptomycin (P/S; Sigma‐Aldrich, USA), AMX (Wako, Japan), azithromycin dihydrate (AZM; Tokyo Chemical Industry, Japan), cefdinir (CDR; Sigma‐Aldrich), minocycline HCl (MIN; LKT Laboratories, USA), and MTZ (LKT Laboratories). All antibiotics, except P/S, were used at concentration of 1 mg/L. This concentration is determined based on the maximum serum concentration after oral administration at clinical use provided by each pharmaceutical manufacture (http://database.japic.or.jp). In addition, for better comparison of each antibiotic activity against injected bacteria, all antibiotics were used at the same concentration although minimum inhibitory concentration against each microorganism may slightly differ. P/S was used at higher concentration throughout the experiments so that P/S would serve as a positive control (100 IU penicillin and 100 mg/L streptomycin). This high concentration is generally used for isolating eukaryotic cells from various tissues. To verify the effects of combination, for the combination of AMX + MTZ and CDR + MTZ, each dose was used at 1 mg/L, and therefore, the final concentration became 2 mg/L.

### 2.2 Liquid culture antibiotic assay

To verify effects of antibiotics on each strain, 100 μl of culture medium containing each strain was added to 3 ml of α‐MEM containing 10% FBS, with or without test antibiotics, and incubated for 24 hr at 37 °C under hypoxic conditions (5% CO_2_, 2% O_2_, and 93% N_2_) in a multi‐gas incubator. For verification of effects of antibiotics on combinations of two different bacterial strains, 50 μl of each culture medium containing test microorganism was mixed, and a total of 100 μl of test samples was added to 3 ml of α‐MEM medium, with or without test antibiotics.

**Figure 1:**
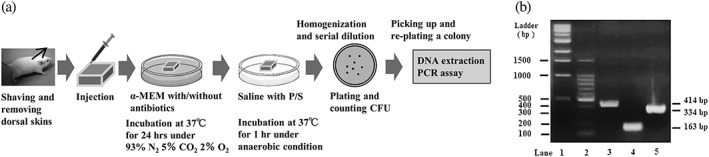
Schematic presentation of the organ culture antibiotic assay (a) and amplification of DNA from test bacteria by polymerase chain reaction (b). (a) Test mice were sacrificed, and dorsal skin was removed after gentle shaving. Tissue was trimmed to a 5‐mm square and washed using the medium as described in Section 2. One hundred microliter of α‐MEM with 10% fetal bovine serum (FBS) containing bacterial samples was injected into washed organs using 32‐gage needle syringes. These tissues containing bacterial samples were incubated in α‐MEM containing test antibiotics supplemented with 10 % FBS at 37 °C for 24 hr under hypoxic conditions (5% CO_2_, 2% O_2_, and 93% N_2_) in a multi‐gas incubator. Tissue was then washed with phosphate‐buffered saline (PBS) 24 hr later. To completely remove surface bacteria, they were soaked in 3 ml of PBS containing 100 IU/100 mg/L penicillin/streptomycin solution at 37 °C for 1 hr. Then, this tissue was homogenized using a homogenizer, and tissue homogenates were serially diluted. The samples were transferred onto blood agar plates, followed by the counting of colony‐forming units. Then, the bacterial colonies were subjected to polymerase chain reaction (PCR) assay. (b) The validity of PCR assay for an identification of test microorganisms was confirmed prior to the experiments. Lanes 1 and 2: molecular marker (ladder); lane 2: *Porphyromonas gingivalis*; lane 3: *Prevotella intermedia*; and lane 4: *Fusobacterium nucleatum.* CFU, colony‐forming unit

Twenty‐four hours later, culture medium was centrifuged of 7000 *g* at 4 °C for 7 min, and the pellet was washed with phosphate‐buffered saline (PBS) and serially diluted to measure CFUs. One hundred microliters of the samples was transferred onto blood agar plates containing 5 mg/L hemin, 10 mg/L menadione, 400 mg/L L‐cysteine, and 5% sheep blood (CDC anaerobic culture plate, NACALAI TESQUE) and incubated for 5 days, followed by counting CFU. After that procedure, a single colony was picked up and transferred onto an agar plate again and incubated for additional 2–5 days. These colonies were used for an identification of target bacteria by polymerase chain reaction (PCR). Bacterial DNA was isolated using InstaGene Matrix (Bio‐Rad Laboratories, USA), according to manufacturer's instructions, and subjected to PCR analysis. Primers used for identification of each bacteria have been described previously as follows (Fujise, Hamachi, Inoue, Miura, & Maeda, 2002; Garcia, Tercero, Legido, Ramos, Alemany, & Sanz, 1998; Kulekci, Ciftci, & Keskin, 2001): *Pg* (414‐bp: GACCTAAAGGCCATCCCGTA for forward, AGCCTCGGTTGAATACCGTA for reverse), *Pi* (163‐bp: CGTGCCAGCCGCGGTAATACG for forward, TCCGCATACGTTGCGTGCACTCAAG for reverse), and *Fn* (334b‐p: CTAAATACGTGCCAGCAGCC for forward, CGACCCCCAACACCTAGTAA for reverse). We performed PCR analysis using Ex‐Taq (TAKARA BIO, Japan) according to the manufacturer's instruction. PCR was performed under the following conditions: (a) an initial denaturation at 94 °C for 30 s; (b) 30 cycles consisting of denaturation at 94 °C for 30 s, annealing at 55 °C for 30 s, and extension at 72 °C for 1 min; and (c) a final extension at 72 °C for 7 min. After amplification, 10 μl of PCR products was subjected to electrophoresis in a 1.2% agarose gel. The DNA samples were stained with 0.5 mg/L ethidium bromide and visualized under ultraviolet light. Validity of PCR amplification was confirmed in advance (Figure 1b). Both *Pg* and *Pi* are black‐pigmented bacteria. To visually distinguish these two bacteria, 366‐nm ultraviolet light was irradiated onto the colonies, and pink/orange colonies were considered to be *Pi*, while black colonies were considered to be *Pg*; this was performed prior to confirmation by PCR, as described previously (Reynolds & Slots, 1982).

### 2.3 Organ culture antibiotic assay

Prior to experimentation, protocols using animals were approved by Kyushu University's Animal Experiments Review Board (Approval numbers: A25‐249‐0 and A27‐115‐0). Schematic presentation for organ culture antibiotic assay was summarize in Figure 1a. Briefly, 8‐week‐old male Sea:ddY mice were used for organ culture experiments. Immediately after sacrifice via intraperitoneal injection of excess amounts of pentobarbital (100 μl of 63.8 mg/ml pentobarbital) and wiping using 70 % ethanol, dorsal skin was removed after gentle shaving using a sterile shaver. A total of 40 mice were used for this study, and 5 or 6 tissues were collected from each mouse. Each tissue was trimmed to a 5‐mm square and washed using α‐MEM containing 10% FBS. As described above, 100 μl of α‐MEM containing bacterial samples was injected into washed organs using 32‐gage needle syringes. These tissues containing bacterial samples were incubated in α‐MEM containing test antibiotics supplemented with 10% FBS at 37 °C for 24 hr under hypoxic conditions (5% CO_2_, 2% O_2_, and 93% N_2_). Tissue was washed with PBS 24 hr later. To completely remove surface bacteria, they were soaked in 3 ml of PBS containing 100 IU/100 mg/L P/S solution at 37 °C for 1 hr. Then, these tissues were homogenized using a hand homogenizer for 1 min, according to manufacturer's instructions (BioMasherII and PowerMasherII, Nippi Incorporated, Japan), and tissue homogenates were serially diluted. One hundred microliter of the samples was transferred onto blood agar plates. Measurement of CFU and identification of colony bacteria by PCR were described above. To confirm the invasion of test bacteria as well as the integrity of the test tissues after *Pg* injection, histological observations using gram staining of test specimens were performed (a) before bacterial injection, (b) immediately after *Pg* injection, (c) 24 hr later following organ culture without *Pg* injection, and (d) 24 hr after *Pg* injection.

### 2.4 Statistical analysis

All experiments were performed by triplicate. Statistical analyses were performed using analysis of variance. Once statistical significance on analysis of variance was confirmed, post hoc pairwise comparisons were conducted using Tukey's multiple comparison; *p* < .05 was considered statistically significant.

## 3 RESULTS

### 3.1 Colony‐forming ability of bacteria after antibiotic treatment in liquid medium

Results of each antibiotics and combination of antibiotics to a single bacterium or co‐cultures of two bacteria in α‐MEM containing FBS are shown in Table 1. All antibiotics appeared to be effective against target bacteria with some diverse activities. No remarkable changes were observed in terms of antibiotic activity when test bacteria were mixed and co‐cultured.

**Table 1:** Antibiotic effects assessed by the colony‐forming ability of test antibiotics against single strain infection or two co‐infections in 24 hr liquid medium

	Bacteria strains of single infection or two strains co‐infections (mean log_10_ CFU ± SEM)								
	*Pg* single	*Pg*/*Pi*‐*Pg*	*Pg*/*Fn*‐*Pg*	*Pi* single	*Pg*/*Pi*‐*Pi*	*Pi*/*Fn*‐*Pi*	*Fn* single	*Pg*/*Fn*‐*Fn*	*Pi*/*Fn*‐*Fn*
No Antibiotics	9.01 ± 0.19	9.10 ± 0.09	9.52 ± 0.56	8.77 ± 0.46	8.75 ± 0.38	8.42 ± 0.16	6.53 ± 0.26	6.96 ± 0.27	6.28 ± 0.48
AMX	2.53 ± 0.22	1.91 ± 0.14	1.79 ± 0.19	1.03 ± 0.07	1.22 ± 0.10	1.33 ± 0.19	1.84 ± 0.26	1.87 ± 0.24	2.03 ± 0.11
AZM	ND	1.01 ± 0.39	1.64 ± 0.30	ND	1.44 ± 0.63	2.04 ± 0.40	3.51 ± 0.23	3.11 ± 0.15	3.18 ± 0.14
CDR	2.43 ± 0.28	1.80 ± 0.15	1.67 ± 0.23	1.95 ± 0.21	2.17 ± 0.14	2.04 ± 0.21	2.34 ± 0.17	2.19 ± 0.21	2.29 ± 0.17
MIN	3.68 ± 0.07	3.44 ± 0.17	3.29 ± 0.18	3.31 ± 0.05	3.49 ± 0.13	3.30 ± 0.39	3.61 ± 0.14	3.46 ± 0.10	2.91 ± 0.28
MTZ	2.72 ± 0.35	2.78 ± 0.64	2.93 ± 0.40	2.39 ± 0.18	2.67 ± 0.33	2.38 ± 0.17	3.43 ± 0.05	3.33 ± 0.04	3.38 ± 0.09
AMX +MTZ	1.56 ± 0.27	1.57 ± 0.58	1.73 ± 0.63	0.85 ± 0.33	1.57 ± 0.58	1.37 ± 0.51	1.56 ± 0.27	1.73 ± 0.63	1.37 ± 0.51
CDR +MTZ	1.23 ± 0.47	0.60 ± 0.33	0.60 ± 0.33	ND	0.87 ± 0.43	1.15 ± 0.53	1.14 ± 0.44	ND	1.31 ± 0.49
P/S	ND	ND	ND	ND	ND	ND	ND	ND	ND

*Note*. *Pg/Pi*‐*Pg*, *Pg* CFU of *Pg* and *Pi* co‐infection, *Pg/Pi*‐*Pi*, *Pi* CFU of *Pg* and *Pi* co‐infection, *Pg/Fn*‐*Pg*, *Pg* CFU of *Pg* and *Fn* co‐infection, *Pg/Fn*‐*Fn*, *Fn* CFU of *Pg* and *Fn* co‐infection, *Pi/Fn*‐*Pi*, *Pi* CFU of *Pi* and *Fn* co‐infection, and *Pi/Fn*‐*Fn*, *Fn* CFU of *Pi* and *Fn* co‐infection.

AMX = amoxicillin; AZM = azithromycin = CDR, cefdinir; CFU = colony forming units; *Fn* = *Fusobacterium nucleatum*; MIN = minocycline; MTZ = metronidazole; ND = not detected; *Pg* = *Porphyromonas gingivalis*; *Pi* = *Prevotella intermedia*; P/S = penicillin/streptomycin.

### 3.2 Colony‐forming ability by antibiotics in organ culture infection models

**Figure 2:**
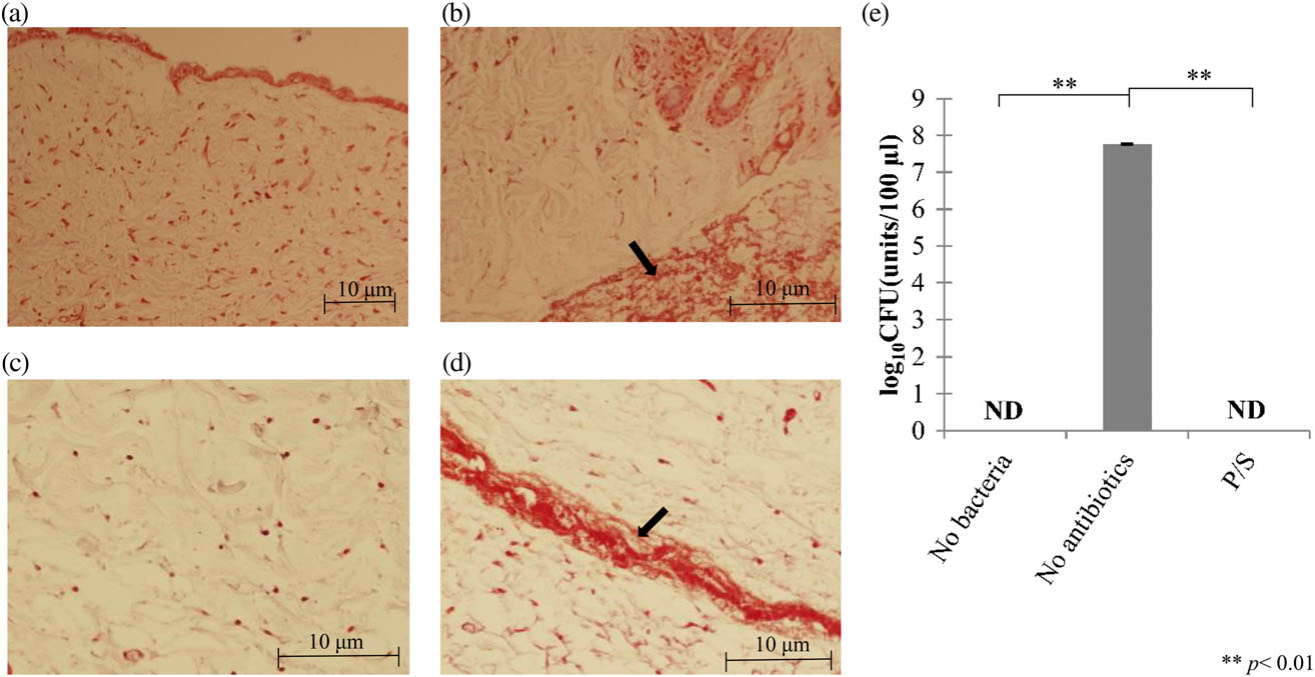
Gram staining of the tissue sections (a–d) and colony‐forming unit (CFU) of test samples injected no bacteria (e – left), injected by *Porphyromonas gingivalis* (*Pg*) but no antibiotics (e – middle), and injected with *Pg* and incubated with penicillin/streptomycin (e – right) in organ culture. (a) Gram staining appearance of epithelia without *Pg* injection (low magnification), and (b) with *Pg* injection; (c) after 24 hr incubation without *Pg* injection; and (d) with *Pg* injection. Black arrow indicates bacterial invasion area. (e) Verification of organ culture procedure. Log_10_ CFU of test samples were injected with no bacteria (e – left), or with *Pg* but no antibiotics (e – middle), and with *Pg* treated by penicillin/streptomycin (e – right). After 24 hr organ culture, all samples were treated with phosphate‐buffered saline containing 100 IU/100 mg/L penicillin/streptomycin solution at 37 °C for 1 hr to remove surface bacteria. The bars represent mean ± SEM of triplicate experiments for each group. **p < .01 by analysis of variance/Tukey test

Before performing organ culture assays, we tested the validity of our protocol by using *Pg* as the test bacteria. We first verified the invasion of bacteria inside the test epithelium. Tissues incubated for 0 or 24 hr, with or without *Pg* injections, were paraffin embedded and gram stained. As shown in Figure 2, *Pg*‐injected tissues were positively stained by gram staining 24 hr after incubation without antibiotics (Figure 2d), while in control sections, positively stained bacteria were not observed (Figure 2c). Tissue integrity appeared to be well maintained in 24 hr as far as judged by histological observations when we compare each figure (Figure 2a–d). We also tried to confirm the colony‐forming ability of the samples obtained from the tissues that corresponded to (a) the one not injected with *Pg* (Figure 2e: no bacteria), (b) the one injected with *Pg* but not treated with any antibiotics (Figure 2e: no antibiotics), and (c) the one injected with *Pg* and incubated with P/S (Figure 2e: P/S). Importantly, no other bacterial contaminations were observed throughout these approaches. Therefore, we proceeded to the next step.

Next, we evaluated the effects of antibiotics in organ culture model. Results of each antibiotics and combination of antibiotics to single bacterial infection or mixed organ culture models are shown in Table 2. CDR and MTZ appeared more effective than AZM and MIN against *Pg* in single‐infection organ culture, while AZM and CDR were more effective than MIN and MTN against *Pi*. CDR was most effective against *Fn*.

**Table 2:** Antibiotic effects assessed by the colony‐forming ability of test antibiotics against single strain infection or two co‐infections in 24 hr organ cultures

	Bacteria strains of single infection or two strains co‐infections (mean log_10_ CFU ± SEM)								
	*Pg* single	*Pg*/*Pi*‐*Pg*	*Pg*/*Fn*‐*Pg*	*Pi* single	*Pg*/*Pi*‐*Pi*	*Pi*/*Fn*‐*Pi*	*Fn* single	*Pg*/*Fn*‐*Fn*	*Pi*/*Fn*‐*Fn*
No Antibiotics	7.76 ± 0.02	7.92 ± 0.24	7.92 ± 0.15	7.72 ± 0.33	7.65 ± 0.07	7.33 ± 0.11	7.13 ± 0.81	6.79 ± 0.62	7.55 ± 0.38
AMX	3.11 ± 0.02	3.13 ± 0.03	3.31 ± 0.02	1.92 ± 0.05	1.67 ± 0.61	1.67 ± 0.61	1.12 ± 0.10	3.07 ± 0.41	1.83 ± 0.66
AZM	4.67 ± 0.50	5.50 ± 0.31	5.44 ± 0.30	2.87 ± 0.19	5.61 ± 0.16	2.17 ± 0.46	4.98 ± 0.16	4.51 ± 0.19	3.03 ± 0.27
CDR	3.21 ± 0.11	3.80 ± 0.25	4.50 ± 0.09	2.12 ± 0.33	2.64 ± 0.94	2.15 ± 0.34	2.71 ± 0.08	4.64 ± 0.22	2.19 ± 0.35
MIN	5.23 ± 0.07	5.25 ± 0.08	5.37 ± 0.45	5.86 ± 0.45	5.86 ± 0.24	4.13 ± 0.74	5.46 ± 0.36	4.75 ± 0.33	5.04 ± 0.33
MTZ	2.87 ± 0.10	3.48 ± 0.29	2.84 ± 0.67	4.43 ± 0.27	4.57 ± 0.09	4.44 ± 0.66	5.43 ± 0.40	3.07 ± 0.04	4.83 ± 0.04
AMX +MTZ	1.22 ± 0.10	1.64 ± 0.26	1.37 ± 0.51	1.37 ± 0.51	1.37 ± 0.51	ND	0.85 ± 0.33	1.01 ± 0.39	ND
CDR +MTZ	1.01 ± 0.39	1.43 ± 0.20	1.48 ± 0.55	1.14 ± 0.43	2.07 ± 0.37	ND	ND	ND	ND
P/S	ND	ND	ND	ND	ND	ND	ND	2.33 ± 0.18	ND

*Note*. *Pg/Pi*‐*Pg*, *Pg* CFU of *Pg* and *Pi* co‐infection, *Pg/Pi*‐*Pi*, *Pi* CFU of *Pg* and *Pi* co‐infection, *Pg/Fn*‐*Pg*, *Pg* CFU of *Pg* and *Fn* co‐infection, *Pg/Fn*‐*Fn*, *Fn* CFU of *Pg* and *Fn* co‐infection, *Pi/Fn*‐*Pi*, *Pi* CFU of *Pi* and *Fn* co‐infection, and *Pi/Fn*‐*Fn*, *Fn* CFU of *Pi* and *Fn* co‐infection.

AMX = amoxicillin; AZM = azithromycin; CDR = cefdinir; CFU = colony forming units; *Fn* = *Fusobacterium nucleatum*; MIN = minocycline; MTZ = metronidazole; ND = not detected; *Pg* = *Porphyromonas gingivalis*; *Pi* = *Prevotella intermedia*; P/S = penicillin/streptomycin.

**Figure 3:**
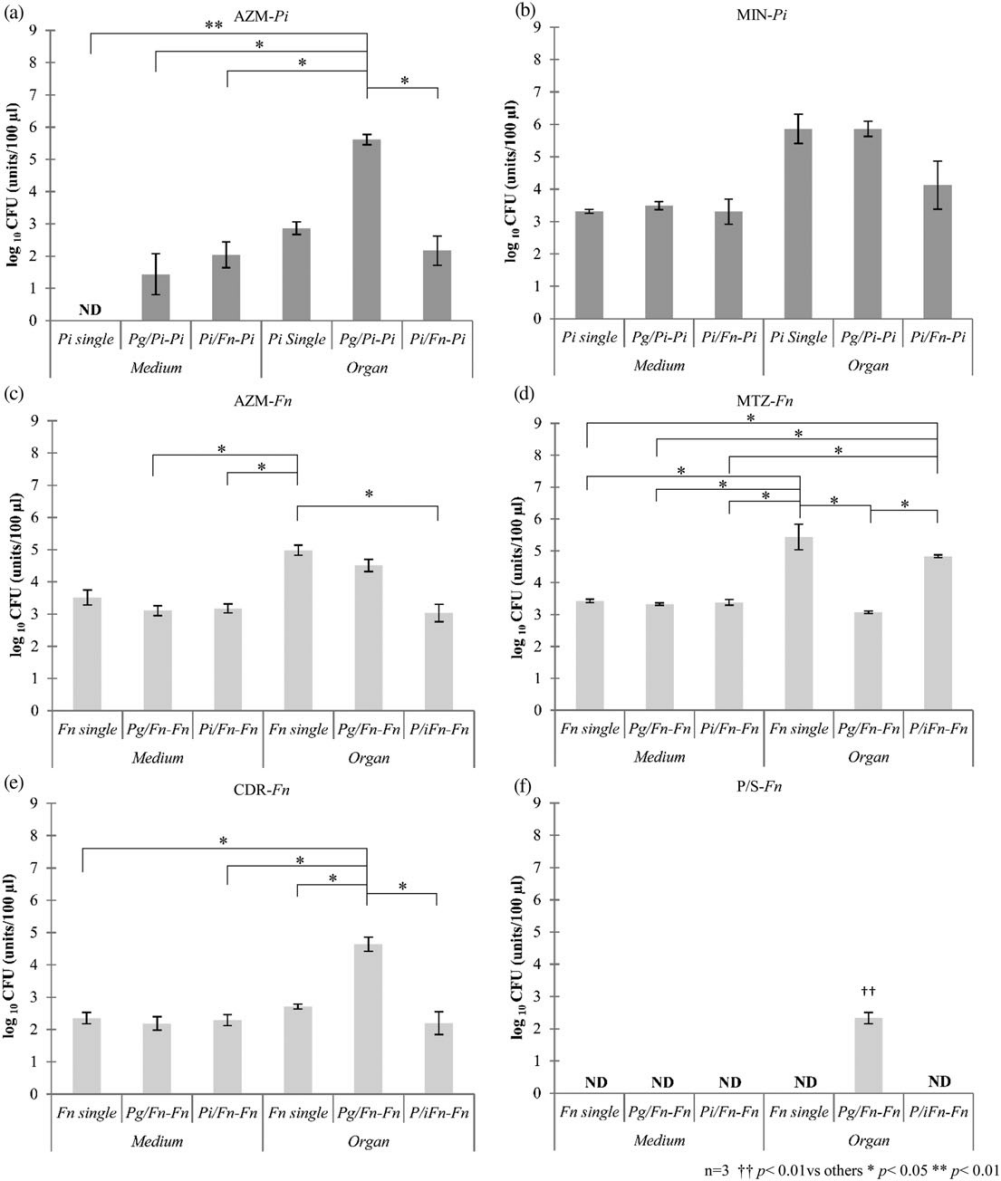
Diverse antibiotic effect of test antibiotics in liquid medium or organ cultures against single or co‐infection model, (a) azithromycin to *Prevotella intermedia* (*Pi*); (b) minocycline to *Pi*; (c) azithromycin to *Fusobacterium nucleatum* (*Fn*); (d) metronidazole to *Fn*; (e) cefdinir to *Fn*; and (f) penicillin/streptomycin to *Fn*. Antibiotic effects with significant changes were picked up and summarized. *Pg*/*Pi*‐*Pi*: *Pi* colony‐forming unit (CFU) of *Porphyromonas gingivalis* (*Pg*) and *Pi* co‐infection, *Pg*/*Fn*‐*Fn: Fn* CFU of *Pg* and *Fn* co‐infection, *Pi*/*Fn*‐*Pi: Pi* CFU of *Pi* and *Fn* co‐infection, and *Pi*/*Fn*‐*Fn: Fn* CFU of *Pi* and *Fn* co‐infection. Penicillin/streptomycin was used at 100 IU/100 mg/L, while other antibiotics were used at 1 mg/L. The bars represent mean ± SEM of triplicate experiments for each group. *p < .05, and **p < .01 represents significantly different result from indicated group. ^††^ *p* < .01 represents significantly different results from the other groups (analysis of variance/Tukey test)

Surprisingly, the results of co‐infections organ culture were different from single‐infection organ culture experiments, and among each group of combination patterns, results also differed. Figure 3 shows the overall results of the effects of antibiotics that changed dramatically when organ culture models were utilized. In the *Pg* and *Pi* co‐incubation model, the antibiotic effect of AZM on *Pi* appeared markedly suppressed, although AZM was very effective in the single infection model (Figure 3a). In *Pi* and *Fn* co‐incubation model, antibiotic effect of MIN against Pi appeared slightly enhanced, although no statistically significant difference was observed (Figure 3b). On the other hand, in the *Pi* and *Fn* co‐incubation model, the antibiotic effect of AZM on *Fn* dramatically increased, although AZM showed less effect on the *Pg* and *Fn* co‐infection model and on the *Fn* single infection model (Figure 3c). In the *Pg* and *Fn* co‐incubation model, the antibiotic effects of P/S and CDR on *Fn* markedly decreased, although both sufficiently inhibited the growth of *Fn* in the single‐infection organ culture model (Figure 3e and f), while MTZ exhibited relatively good responses against *Fn* when these bacteria were co‐incubated with *Pg* in organ culture (Figure 3d).

## 4 DISCUSSION

In our study, we established periodontal inner epithelial infection models using mice dorsal skin. The benefit of this approach is summarized as follows: (a) Histological structures of two different tissues are relatively similar, and mice tissue is easier to handle (not too small to handle and inject test microorganisms) and (b) P/S mixture, which is used for eukaryotic cell cultures, almost completely suppressed growth of resident microorganisms on the surface of mice skin with 1‐hr treatment following organ culture. We initially performed preliminary experiments to eliminate surface bacteria. However, unfortunately, antibiotics appeared to reside inside the tissues, and injected bacteria could not survive. Therefore, we chose P/S treatment after the incubation. According to our preliminary experiments, 30‐min treatment by P/S was not enough to completely remove surface bacteria, while treatment over 2 hr resulted in the disappearance of test bacteria. Thus, after repeated trial, we confirmed 1‐hr treatment was adequate to avoid unwanted effect by P/S.

Throughout the experiments, organ culture was performed for 24 hr. One could argue, if we wish to reproduce chronic periodontal conditions, the incubation time may not be long enough. However, we observed the decay of test tissues after 36–48 hr incubation. In addition, clear bactericidal effects by antibiotics were not observed when incubation time was less than 24 hr. Based on these preliminary experiments, we chose 24 hr incubation. Because we could not observe any inflammatory cell infiltration after 24 hr incubation, the bactericidal effects might be mediated by antibiotics inside the tissues by osmotic action in our organ culture model, but not by immunological action.

As test microorganisms, we chose *Pg*, *Pi*, and *Fn* for our current experiments. *Pg* is an important microorganism in the deep pockets of the subjects with severe adult chronic periodontitis and is a member of the “red complex” family, which is closely associated with the etiology of the disease (Haffajee & Socransky, 2002). Although both *Pi* and *Fn* are not members of the “red complex” family—they belong to the “orange complex” family —it has been reported that *Fn* plays a very important role in biofilm formation by adhering to *Pg* and *Pi*, both of which are late colonizers (Kolenbrander et al., 2002). Therefore, we hypothesize that the existence of *Fn* might greatly influence antibiotic susceptibility. As expected, results obtained from organ culture experiments, especially those using mixed infection models, greatly differed from results from regular liquid culture models. It has been reported that *Pi* may enhance antibiotic resistance when co‐cultured with other bacteria (Takahashi et al., 2006). However, at least in our current study, similar effects were not observed. Therefore, the involvement of *Pi* in the changes of antibiotic resistance needs to be further examined. In this study, antibiotics are more ineffective against *Fn* in organ culture model. *Fn* is a key player in biofilm formation. However, it is still unclear whether these bacteria invade host tissue or not. If we assume *Fn* invade the tissues, antibiotics would be ineffective against *Fn*. In this study, combination of two bacteria was used for organ culture model. We also tried to see the effects of antibiotics on mixed multiculture model of three bacterial strains. However, in that case, *Pi* growth was not observed (data not shown). According to the previous study, *Pi* did not sufficiently grow in studying in vitro biofilm‐forming model (Soares et al., 2015). Further studies are needed to fully elucidate this point.

Azithromycin was not as successful as other antibiotics tested in organ culture model. Some clinical reports indicated the similar results (Han et al., 2012, Haas et al., 2012). With respect to colony‐forming ability of the samples obtained from organ culture infection models treated with test antibiotics, the combination of AMX or CDR and MTZ effectively treated all bacterial combinations that we tested (Table 2). Interestingly, CDR, not AMX, is frequently used in Japan due to less chances of inducing penicillin shock (Pichichero and Pichichero, 1998). Therefore, CDR is considered to be a counterpart to AMX. In our experiments using AMX and CDR, CDR even exhibited almost equal or even superior effects. From a clinical standpoint, combination of both MTZ and AMX with conventional mechanical debridement has been shown to be effective as a treatment for severe periodontitis (Silva et al., 2011), especially aggressive periodontitis (Rodrigues et al., 2012; Sgolastra et al., 2012), which is characterized by early‐onset and rapid disease progression (Schenkein & Van Dyke, 1994). Although the etiology of aggressive periodontitis is still unclear, these subjects may be characterized by some defects in host defense system due to rapid disease progression (Kulkarni, & Kinane 2014). In fact, late‐stage human immunodeficient virus‐infected subjects and/or severe neutropenia are associated with severe periodontitis (Deas, Mackey, & McDonnell, 2003; Hajishengallis & Hajishengallis, 2014; Lucht, Heimdahl, & Nord, 1991). Because host‐defensive functions are severely impaired in these subjects, it is quite possible that substantial amounts of periodontal bacteria actually invade inflamed periodontal tissue, and the legion of subjects with aggressive periodontitis may also harbor such bacteria.

As described previously, combinations of local antibiotics with conventional mechanical treatment resulted in greater reductions in circulating inflammatory markers and subsequent glycated hemoglobin levels in subjects with type 2 diabetes (Munenaga et al., 2013). These subjects are also considered to be more or less immunocompromised. Therefore, it is important to establish sophisticated therapeutic strategies that diminish systemic inflammation in these subjects. If combination of antibiotics with the conventional therapy for subjects with severe periodontitis results in improvement of clinical parameters and systemic inflammation, use of antibiotics will be extremely important in such subjects. Interestingly, recently, combination of AMX and MTZ was shown to be effective against artificially established multibiofilm model (Soares et al., 2015). We believe that our current results would support the results of this study from another aspect. Taken together, it is interesting to compare the effects of the combination of these antibiotics plus mechanical debridement on the resolution of systemic inflammation with conventional mechanical debridement alone by clinical intervention study.

## References

[cre248-bib-0001] Almaghlouth, A. A. , Cionca, N. , Cancela, J. A. , Decaillet, F. , Courvoisier, D. S. , Giannopoulou, C. , & Mombelli, A. (2014). Effect of periodontal treatment on peak serum levels of inflammatory markers. Clin Oral Investig, 18, 2113–2121. 10.1007/s00784-014-1187-4 24452825

[cre248-bib-0002] Andres, M. T. , Chung, W. O. , Roberts, M. C. , & Fierro, J. F. (1998). Antimicrobial susceptibilities of *Porphyromonas gingivalis*, *Prevotella intermedia*, and *Prevotella nigrescens spp*. isolated in Spain. Antimicrob Agents Chemother, 42, 3022–3023. 9797247 10.1128/aac.42.11.3022 PMC105987

[cre248-bib-0003] Bolstad, A. I. , Jensen, H. B. , & Bakken, V. (1996). Taxonomy, biology, and periodontal aspects of *Fusobacterium nucleatum* . Clin Microbiol Rev, 9, 55–71. 8665477 10.1128/cmr.9.1.55 PMC172882

[cre248-bib-0004] Borgnakke, W. S. , Chapple, I. L. C. , Genco, R. J. , Armitage, G. , Bartold, P. M. , D'Aiuto, F. , et al. (2014). The multi‐center randomized controlled trial (RCT) published by the Journal of the American Medical Association (JAMA) on the effect of periodontal therapy on glycated hemoglobin (HbA_1c_) has fundamental problems. J Evid based Dent Pract., 3, 127–132. 10.1016/j.jebdp.2014.04.017 PMC4502578 25234213

[cre248-bib-0005] Cionca, N. , Giannopoulou, C. , Ugolotti, G. , & Mombelli, A. (2009). Amoxicillin and metronidazole as an adjunct to full‐mouth scaling and root planing of chronic periodontitis. J Periodontol., 80, 364–371. 19254119 10.1902/jop.2009.080540

[cre248-bib-0006] Deas, D. E. , Mackey, S. A. , & McDonnell, H. T. (2003). Systemic disease and periodontitis: Manifestations of neutrophil dysfunction. Periodontol 2000, 32, 82–104. 12756035 10.1046/j.0906-6713.2003.03207.x

[cre248-bib-0007] Ehmke, B. , Moter, A. , Beikler, T. , Milian, E. , & Flemmig, T. F. (2005). Adjunctive antimicrobial therapy of periodontitis: Long‐term effects on disease progression and oral colonization. J Periodontol., 76, 749–759. 15898936 10.1902/jop.2005.76.5.749

[cre248-bib-0008] Feres, M. , Soares, G. M. , Mendes, J. A. , Silva, M. P. , Faveri, M. , Teles, R. , … Figueiredo, L. C. (2012). Metronidazole alone or with amoxicillin as adjuncts to non‐surgical treatment of chronic periodontitis: A 1‐year double‐blinded, placebo‐controlled, randomized clinical trial. J Clin Periodontol., 39, 1149–1158. 23016867 10.1111/jcpe.12004

[cre248-bib-0009] Fosse, T. , Madinier, I. , Hannoun, L. , Giraud‐Morin, C. , Hitzig, C. , Charbit, Y. , & Ourang, S. (2002). High prevalence of cfxA beta‐lactamase in aminopenicillin‐resistant *Prevotella* strains isolated from periodontal pockets. Oral Microbiol Immunol., 17, 85–88. 11929554 10.1046/j.0902-0055.2001.00096.x

[cre248-bib-0010] Fujise, O. , Hamachi, T. , Inoue, K. , Miura, M. , & Maeda, K. (2002). Microbiological markers for prediction and assessment of treatment outcome following non‐surgical periodontal therapy. J Periodontol, 73, 1253–1259. 12479627 10.1902/jop.2002.73.11.1253

[cre248-bib-0011] Garcia, L. , Tercero, J. C. , Legido, B. , Ramos, J. A. , Alemany, J. , & Sanz, M. (1998). Rapid detection of *Actinobacillus actinomycetemcomitans*, *Prevotella intermedia* and *Porphyromona*s *gingivalis* by multiplex PCR. J Periodontal Res, 33, 59–64. 9524322 10.1111/j.1600-0765.1998.tb02292.x

[cre248-bib-0012] Giannopoulou, C. , Cionca, N. , Almaghlouth, A. , Cancela, J. , Courvoisier, D. S. , & Mombelli, A. (2016). Systemic biomarkers in 2‐phase antibiotic periodontal treatment: A randomized clinical trial. J Dent Res., 95, 349–355. 26604272 10.1177/0022034515618949

[cre248-bib-0013] Goodson, J. M. , Haffajee, A. D. , Socransky, S. S. , Kent, R. , Teles, R. , Hasturk, H. , … Lindhe, J. (2012). Control of periodontal infections: A randomized controlled trial I. The primary outcome attachment gain and pocket depth reduction at treated sites. J Clin Periodontol, 39, 526–536. 22512461 10.1111/j.1600-051X.2012.01870.x

[cre248-bib-0014] Haas, A. N. , Silva‐Boghossian, C. M. , Colombo, A. P. , Susin, C. , Albandar, J. M. , Oppermann, R. V. , & Rosing, C. K. (2012). Adjunctive azithromycin in the treatment of aggressive periodontitis: Microbiological findings of a 12‐month randomized clinical trial. J Dent., 40, 556–563. 22445846 10.1016/j.jdent.2012.03.004

[cre248-bib-0015] Hajishengallis, E. , & Hajishengallis, G. (2014). Neutrophil homeostasis and periodontal health in children and adults. J Dent Res, 93, 231–237. 24097856 10.1177/0022034513507956 PMC3929973

[cre248-bib-0016] Han, B. , Emingil, G. , Ozdemir, G. , Tervahartiala, T. , Vural, C. , Atilla, G. , … Sorsa, T. (2012). Azithromycin as an adjunctive treatment of generalized severe chronic periodontitis: Clinical, microbiologic, and biochemical parameters. J Periodontol, 83, 1480–1491. 22324488 10.1902/jop.2012.110519

[cre248-bib-0017] Holt, S. C. , Kesavalu, L. , Walker, S. , & Genco, C. A. (1999). Virulence factors of *Porphyromonas gingivalis* . Periodontol 2000, 20, 168–238. 10522227 10.1111/j.1600-0757.1999.tb00162.x

[cre248-bib-0018] Jeffcoat, M. K. , & Reddy, M. S. (1991). Progression of probing attachment loss in adult periodontitis. J Periodontol., 62, 185–189. 2027069 10.1902/jop.1991.62.3.185

[cre248-bib-0019] Kolenbrander, P. E. , Andersen, R. N. , Blehert, D. S. , Egland, P. G. , Foster, J. S. , & Palmer, R. J. (2002). Communication among oral bacteria. Microbiol Mol Biol R., 66, 486–505. 10.1128/MMBR.66.3.486-505.2002 PMC120797 12209001

[cre248-bib-0020] Kulekci, G. , Ciftci, S. , & Keskin, F. (2001). PCR analysis of *Actinobacillus actinomycetemcomitans*, *Porphyromonas gingivalis*, *Treponema denticola* and *Fusobacterium nucleatum* in middle ear effusion. Anaerobe, 7, 241–246.

[cre248-bib-0021] Kulkarni, C. , & Kinane, D. F. (2014). Host response in aggressive periodontitis. Periodontol 2000, 65, 79–91. 24738587 10.1111/prd.12017

[cre248-bib-0022] Lamont, R. J. , & Yilmaz, O. (2002). In or out: The invasiveness of oral bacteria. Periodontol 2000, 30, 61–69. 12236896 10.1034/j.1600-0757.2002.03006.x

[cre248-bib-0023] Lu, H. K. , & Chei, C. J. (2005). Efficacy of subgingivally applied minocycline in the treatment of chronic periodontitis. J Periodontal Res., 40, 20–27. 15613075 10.1111/j.1600-0765.2004.00763.x

[cre248-bib-0024] Lucht, E. , Heimdahl, A. , & Nord, C. E. (1991). Periodontal disease in HIV‐infected patients in relation to lymphocyte subsets and specific micro‐organisms. J Clin Periodontol., 18, 252–256. 1677365 10.1111/j.1600-051x.1991.tb00423.x

[cre248-bib-0025] Miranda, T. S. , Feres, M. , Perez‐Chaparro, P. J. , Faveri, M. , Figueiredo, L. C. , Tamashiro, N. S. , … Duarte, P. M. (2014). Metronidazole and amoxicillin as adjuncts to scaling and root planing for the treatment of type 2 diabetic subjects with periodontitis: 1‐year outcomes of a randomized placebo‐controlled clinical trial. J Clin Periodontol., 41, 890–899. 24930639 10.1111/jcpe.12282

[cre248-bib-0026] Moutsopoulos, N. M. , & Madianos, P. N. (2006). Low‐grade inflammation in chronic infectious diseases: Paradigm of periodontal infections. Ann NY Acad Sci., 1088, 251–264. 17192571 10.1196/annals.1366.032

[cre248-bib-0027] Munenaga, Y. , Hiroshima Study, G. , Yamashina, T. , Tanaka, J. , & Nishimura, F. (2013). Improvement of glycated hemoglobin in Japanese subjects with type 2 diabetes by resolution of periodontal inflammation using adjunct topical antibiotics: Results from the Hiroshima Study. Diabetes Res Clin Pr., 100, 53–60. 10.1016/j.diabres.2013.01.028 23465365

[cre248-bib-0028] Page, R. C. , Offenbacher, S. , Schroeder, H. E. , Seymour, G. J. , & Kornman, K. S. (1997). Advances in the pathogenesis of periodontitis: Summary of developments, clinical implications and future directions. Periodontol 2000, 14, 216–248. 9567973 10.1111/j.1600-0757.1997.tb00199.x

[cre248-bib-0029] Pichichero, M. E. , & Pichichero, D. M. (1998). Diagnosis of penicillin, amoxicillin, and cephalosporin allergy: Reliability of examination assessed by skin testing and oral challenge. J Pediatr‐Us., 132, 137–143. 10.1016/s0022-3476(98)70499-8 9470015

[cre248-bib-0030] Rabbani, G. M. , Ash, M. M. Jr. , & Caffesse, R. G. (1981). The effectiveness of subgingival scaling and root planing in calculus removal. J Periodontol., 52, 119–123. 7014822 10.1902/jop.1981.52.3.119

[cre248-bib-0031] Rodrigues, A. S. , Lourencao, D. S. , Lima Neto, L. G. , Pannuti, C. M. , Hirata, R. D. , Hirata, M. H. , … De Micheli, G. (2012). Clinical and microbiologic evaluation, by real‐time polymerase chain reaction, of non‐surgical treatment of aggressive periodontitis associated with amoxicillin and metronidazole. J Periodontol., 83, 744–752. 22060046 10.1902/jop.2011.110333

[cre248-bib-0032] Schenkein, H. A. , & Van Dyke, T. E. (1994). Early‐onset periodontitis: Systemic aspects of etiology and pathogenesis. Periodontol 2000, 6, 7–25. 9673167 10.1111/j.1600-0757.1994.tb00023.x

[cre248-bib-0033] Sgolastra, F. , Petrucci, A. , Gatto, R. , & Monaco, A. (2012). Effectiveness of systemic amoxicillin/metronidazole as an adjunctive therapy to full‐mouth scaling and root planing in the treatment of aggressive periodontitis: A systematic review and meta‐analysis. J Periodontol., 83, 731–743. 22050545 10.1902/jop.2011.110432

[cre248-bib-0034] Silva, M. P. , Feres, M. , Sirotto, T. A. , Soares, G. M. , Mendes, J. A. , Faveri, M. , & Figueiredo, L. C. (2011). Clinical and microbiological benefits of metronidazole alone or with amoxicillin as adjuncts in the treatment of chronic periodontitis: A randomized placebo‐controlled clinical trial. J Clin Periodontol., 38, 828–837. 21762197 10.1111/j.1600-051X.2011.01763.x

[cre248-bib-0035] Slots, J. , & Reynolds, H. S. (1982). Long‐wave UV light fluorescence for identification of black‐pigmented *Bacteroides spp* . J Clinical Microbiol., 16, 1148–1151. 7161378 10.1128/jcm.16.6.1148-1151.1982 PMC272555

[cre248-bib-0036] Soares, G. M. , Teles, F. , Starr, J. R. , Feres, M. , Patel, M. , Martin, L. , & Teles, R. (2015). Effects of azithromycin, metronidazole plus amoxicillin on an in vitro polymicrobial subgingival biofilm model. Antimicrob Agents Chemother., 59, 2791–2798. 25733510 10.1128/AAC.04974-14 PMC4394767

[cre248-bib-0037] Socransky, S. S. , & Haffajee, A. D. (2002). Dental biofilms: Difficult therapeutic targets. Periodontol 2000, 28, 12–55. 12013340 10.1034/j.1600-0757.2002.280102.x

[cre248-bib-0038] Takahashi, N. , Ishihara, K. , Kimizuka, R. , Okuda, K. , & Kato, T. (2006). The effects of tetracycline, minocycline, doxycycline and ofloxacin on *Prevotella intermedia* biofilm. Oral Microbiol Immunol., 21, 366–371. 17064394 10.1111/j.1399-302X.2006.00305.x

[cre248-bib-0039] Takeuchi, H. , Furuta, N. , & Amano, A. (2011). Cell entry and exit by periodontal pathogen via recycling pathway. Commun Integr Biol., 4, 587–589. 22046471 10.4161/cib.4.5.16549 PMC3204137

[cre248-bib-0040] Zandbergen, D. , Slot, D. E. , Cobb, C. M. , & Van der Weijden, F. A. (2013). The clinical effect of scaling and root planing and the concomitant administration of systemic amoxicillin and metronidazole: A systematic review. J Periodontol., 84, 332–351. 22612369 10.1902/jop.2012.120040

